# Harnessing Gene Expression Networks to Prioritize Candidate Epileptic Encephalopathy Genes

**DOI:** 10.1371/journal.pone.0102079

**Published:** 2014-07-09

**Authors:** Karen L. Oliver, Vesna Lukic, Natalie P. Thorne, Samuel F. Berkovic, Ingrid E. Scheffer, Melanie Bahlo

**Affiliations:** 1 Bioinformatics Division, The Walter and Eliza Hall Institute of Medical Research, Melbourne, Victoria, Australia; 2 Epilepsy Research Center, Department of Medicine, University of Melbourne, Austin Health, Heidelberg, Victoria, Australia; 3 Florey Institute, Melbourne, Victoria, Australia; 4 Department of Paediatrics, University of Melbourne, Royal Children's Hospital, Melbourne, Victoria, Australia; 5 Department of Mathematics and Statistics, University of Melbourne, Melbourne, Victoria, Australia; Shenzhen Institutes of Advanced Technology, China

## Abstract

We apply a novel gene expression network analysis to a cohort of 182 recently reported candidate Epileptic Encephalopathy genes to identify those most likely to be true Epileptic Encephalopathy genes. These candidate genes were identified as having single variants of likely pathogenic significance discovered in a large-scale massively parallel sequencing study. Candidate Epileptic Encephalopathy genes were prioritized according to their co-expression with 29 known Epileptic Encephalopathy genes. We utilized developing brain and adult brain gene expression data from the Allen Human Brain Atlas (AHBA) and compared this to data from Celsius: a large, heterogeneous gene expression data warehouse. We show replicable prioritization results using these three independent gene expression resources, two of which are brain-specific, with small sample size, and the third derived from a heterogeneous collection of tissues with large sample size. Of the nineteen genes that we predicted with the highest likelihood to be true Epileptic Encephalopathy genes, two (*GNAO1* and *GRIN2B*) have recently been independently reported and confirmed. We compare our results to those produced by an established *in silico* prioritization approach called Endeavour, and finally present gene expression networks for the known and candidate Epileptic Encephalopathy genes. This highlights sub-networks of gene expression, particularly in the network derived from the adult AHBA gene expression dataset. These networks give clues to the likely biological interactions between Epileptic Encephalopathy genes, potentially highlighting underlying mechanisms and avenues for therapeutic targets.

## Introduction

The Epileptic Encephalopathies are a clinically and etiologically heterogeneous group of devastating infantile and childhood-onset epilepsies, broadly characterized by refractory seizures and developmental slowing or regression [1]. Following the seminal discovery of *de novo SCN1A* mutations as the cause in >80% of patients with Dravet syndrome [2], a paradigmatic epileptic encephalopathy, a number of other genes have been shown to account for other hitherto unexplained Epileptic Encephalopathies [3]–[6].

Massively parallel sequencing has recently accelerated gene discovery, revealed unexpected genetic heterogeneity and cemented the role played by *de novo* mutations in causing Epileptic Encephalopathies. In particular, Carvill and colleagues [3] recently performed targeted massively parallel re-sequencing of 19 known and 46 candidate genes in 500 Epileptic Encephalopathy cases. They identified pathogenic mutations in 10% of patients in their cohort and established *CHD2* and *SYNGAP1* as novel Epileptic Encephalopathy genes. Another study employed a whole exome sequencing ‘trio design’ of 264 probands with Epileptic Encephalopathies and their parents in search of *de novo* variants [4]. Using a likelihood analysis to evaluate statistical evidence of association, they determined *GABRB3* and *ALG13* to be novel Epileptic Encephalopathy genes. These four genes join *KCNT1* [7] and *GRIN2A* [8]–[10] as new Epileptic Encephalopathy genes and add to the growing number of genes responsible for these devastating disorders.

Along with discovering new ‘definite’ Epileptic Encephalopathy genes, large cohort studies, such as the Epi4K and EPGP Consortia [4] and Carvill et al [3], have also identified variants in many ‘likely’ Epileptic Encephalopathy genes in single subjects. The expectation is that a proportion of these candidate genes will represent true Epileptic Encephalopathy genes, however this determination will require finding additional cases, sufficient to provide statistical evidence of association, and/or supportive functional evidence [11]. The prospect of identifying second and subsequent cases with ‘hits’ in these candidate genes are limited by the very nature that these variants are rare. Moreover the high costs involved in performing functional studies make this impossible for all ‘likely’ Epileptic Encephalopathy genes. Therefore innovative methods are required to identify the “best” candidates on which to focus these follow-up efforts.

There are currently at least 29 ‘definite’ Epileptic Encephalopathy genes. The observation that a number of these known genes are biologically associated (e.g., ion-channel encoding), make it reasonable to hypothesize that true candidate Epileptic Encephalopathy genes will form part of the same or related biological networks. These biological networks are identified and described by looking for evidence of association between genes. This evidence can be gathered using diverse resources including publicly available data such as gene expression, protein-protein interaction (PPI) networks or even literature based searches (i.e., text-mining). Gene networks can be examined and analyzed in their own right to identify modules of co-expression, or as sub-networks, as well as overarching networks with the aid of gene ontology annotation. Another application is their use in an approach known as “guilt by association”, which can be used to prioritize candidate genes according to their level of association with known disease-causing genes (reference set). Alternatively, a genome-wide approach has the potential advantage of identifying novel gene networks, however, it will lack the power gained by utilizing a reference set.

The concept of candidate gene prioritization is well-established and the “guilt by association” principle widely applied [12]–[14]. Many of the current approaches share the same limitations; not least an often heavy reliance on text-mining, which biases against candidate genes with little known about their function and limited published material [12]. Another consideration is that the data sources, utilized by current prioritization methods, typically derive from non-specific resources resulting in ‘generic’ methods that are applied to all disease groups [13]. This is important because data sources are at the core of the gene prioritization problem; the quality of the derived associations directly correlates with the quality of the data used to make these predicted connections. We therefore reasoned that a powerful data source for the prioritization of Epileptic Encephalopathy candidate genes would be gene brain-expression data with the knowledge that gene expression is highly tissue specific. In turn, the large number of known Epileptic Encephalopathy genes (n = 29) allows us to exploit the “guilt by association” principle where these genes will form our reference (or training) set and define our networks. With a focus on brain-expression data the approach will remain unbiased whilst being specific to the Epileptic Encephalopathies as neurological diseases.

The Allen Human Brain Atlas (AHBA) has generated large-scale brain expression data that has been carefully curated and processed, allowing downloading of normalized gene expression data, ready for analysis [15]. The AHBA contains gene expression data from six adult (aged 24–57 years) and four developing (aged 15–21 post-conception weeks) brains. Each brain was dissected carefully and hundreds of arrays were generated for each brain substructure encompassing the whole brain. This contrasts with other gene expression resources where data is typically derived from arbitrarily distributed samples across many individuals. For example, the Celsius resource has gathered thousands of Affymetrix expression data sets from the scientific community into one warehouse. These large sample sizes provide the power to overcome tissue-specific limitations [16] and technical artifacts. UGET (UCLA Gene Expression Tool) is a freely available online tool developed to facilitate data exploration within Celsius [17].

We explore and compare the application of both the AHBA and Celsius expression data to known and candidate Epileptic Encephalopathy gene sets. We use Pearson's and Spearman's correlation coefficient to summarize the linear relationship between gene pairs based on their gene expression data. We also know that negatively correlated genes play an important role in neurological gene networks [18] and a linear relationship fit will allow detection of both positively and negatively correlated genes. Pearson's sample correlation coefficient is the best estimator for the correlation if the underlying data is bivariate normally distributed and the sample size is adequate. However, in the presence of outliers Spearman's correlation coefficient is more robust. In recent years new methods for detecting non-linear relationships have been proposed [19], [20] but initial enthusiasm for these computationally intensive methods has waned, with demonstrations of non-robustness to outliers and the recognition of a severe loss in power if normality and linearity hold approximately for some of these measures [21].

Using methods analogous to leave-one-out cross-validation we show that the known set of 29 Epileptic Encephalopathy genes are substantially co-expressed as a network. We exploit this to prioritize 182 candidate Epileptic Encephalopathy genes resulting from the recent Epi4K and EPGP Consortia study [4]. Empirical false discovery rate (eFDR) thresholds determined the best candidate genes. We argue that these prioritized genes are those most likely to be true Epileptic Encephalopathy genes and merit follow-up studies that may not be possible for all 182 candidates.

## Methods

Statistical analysis and visualization were performed in the statistical programming language R (http://www.r-project.org/) making use of the specific packages gtools [22], qgraph [23], corrplot [24], MASS [25] and reshape [26]. Our methods are implemented in an R package, BrainGEP, and can be downloaded from http://bioinf.wehi.edu.au/software/BrainGEP.

### Expression data sets

#### Allen Human Brain Atlas

We downloaded the normalized microarray gene expression data from the AHBA website (http://www.brain-map.org/) for all six adult and four developing brains. Gene expression data was generated using a custom-made Agilent 8×60K array with 58,692 probes covering 20,782 genes. The design of the array, the normalization procedure and selection of brain samples and dissection protocols are described in whitepapers available from the AHBA website. The probe with the highest median expression value for each gene was chosen to represent the gene expression value for that gene.

#### Celsius

We employed UGET (http://genome.ucla.edu/projects/UGET/) to explore the Celsius gene expression data available for the HG-U133_Plus_2 array design: the largest human dataset with 5,954 CEL files. Approximately 25% of these arrays are of “nervous system origin”, presumably from brain [16]. In the case of genes represented by more than one transcript we chose the longest in base pair length.

### Selection of Epileptic Encephalopathy genes

#### Reference Epileptic Encephalopathy genes

Well-established, known Epileptic Encephalopathy genes were chosen from the literature for our reference set (n = 29); *ALG13*, *ARHGEF9*, *ARX*, *CDKL5*, *CHD2*, *FOXG1*, *GABRA1*, *GABRB3*, *GABRG2*, *GRIN2A*, *HNRNPU*, *KCNQ2*, *KCNT1*, *MBD5*, *MECP2*, *MEF2C*, *PCDH19*, *PLCB1*, *PNKP*, *PNPO*, *SCN1A*, *SCN2A*, *SCN8A*, *SLC2A1*, *SLC25A22*, *SPTAN1*, *STXBP1*, *SYNGAP1* and *UBE3A* (see Table S1).

#### Candidate Epileptic Encephalopathy genes

Candidate genes were selected from a list of genes reported by the Epi4K & EPGP Consortia typically with ‘single hit’ *de novo* variants in their Epileptic Encephalopathy cohort [4]. We limited our selection to 182 genes with variants likely to result in a functional effect (i.e., missense, nonsense, splice-site) (see Table S2 for full list of candidate gene names).

### Detecting co-expression between known and candidate Epileptic Encephalopathy genes

The pairwise Pearson's correlation coefficient (r) for any two genes determined their level of co-expression. We performed these statistical analyses for both AHBA time periods (developing and adult) in R. These analyses were all repeated using Spearman's sample correlation coefficient. We did not have direct access to the Celsius expression data; however, using UGET we were able to generate pairwise Pearson's correlation coefficients for gene pairs.

Pairwise Pearson sample correlation coefficients |r| were calculated for each known Epileptic Encephalopathy gene pair (n = 406). For the AHBA resource, the correlation coefficients were found within each individual, using all brain samples, and then combined within their respective time periods (n = 4 for the developing brain, n = 6 for the adult brains) using a weighting scheme based on the sample variance of the correlation coefficients derived from the gene expression data within each individual. Details can be found in Supporting Information (see Methods S1).

We generated three gene expression correlation matrices (developing AHBA, adult AHBA and Celsius) for all possible gene pairs in the reference set of 29 Epileptic Encephalopathy genes. These results were compared to a list of 1,000 randomly chosen genes (499,500 gene pairs), representing the null distribution, by comparing empirical cumulative distribution functions (ECDFs).

The gene expression correlation matrices for the reference sets were visualized with the corrplot R package [24]. We observed more than one principal component of interest after performing principal component analysis (PCA) on the correlation matrices. We ordered genes based on the angle between the first two principal components, thus summarizing the relationship between them.

### Exploring expression networks between known and candidate Epileptic Encephalopathy genes

A discrete (K*) and continuous (K) connectivity score was generated for each candidate gene (see Methods S1). The connectivity score is a function of the number of significant connections (edges). An edge between a pair of genes is defined as being significant if the Pearson's sample correlation coefficient of pairwise gene expression exceeds a statistically determined threshold. Based on the distribution of all genes available in each dataset we determined the top 5% of |r| cut-off values for each of the three data resources.

Significant correlation networks were generated using the qgraph package [23] in R using Pearson's sample correlation coefficient (r).

### Comparing candidate Epileptic Encephalopathy gene connectivity scores with other predictive resources

We compared connectivity scores between candidates predicted to be pathogenic versus those that were not according to four alternative resources. The chosen resources are a combination of gene and variant-based approaches where we classified results as supporting pathogenicity for each as follows: 1) genic intolerance score (GIT) [28] – genes within the 25^th^ percentile for intolerance, 2) review of the current literature for a gene's prior association with neurological disease, 3) PolyPhen-2 results for gene variants [29] – genes with variants predicted to be damaging (nonsense and splice-site variants classified as damaging), and 4) the recently published CADD (Combined Annotation-Dependent Depletion) [30] resource for variant prioritization – genes with variants determined to have a “scaled” CADD score >25.

For each of the predictive resources of GIT, prior neurological evidence, PolyPhen-2 and CADD predictions, the connectivity scores for candidate genes with evidence for pathogenicity versus those without were compared using one-sided, two-sample Mann-Whitney rank sum tests with continuity correction. P-values were determined using both reference tables and permutation tests where the group membership labels were permuted 1000 times. This was performed for results from the three gene expression resources, and for both the discrete (K*) and continuous (K) connectivity scores.

### *In silico* prioritization of candidate Epileptic Encephalopathy genes

Our method prioritizes candidate genes based on their connectivity scores with known Epileptic Encephalopathy genes. We applied a false discovery rate [31] of 0.25 to determine a connectivity score threshold required for candidates to meet in order to be deemed those most likely true Epileptic Encephalopathy genes. In brief, we derived empirical false discovery rates (eFDR) based on sampling sets of 179 candidate gene sets for both the developing and adult AHBA (3 genes not present on the AHBA array) and 172 candidate genes for the Celsius resource (10 genes not present on the Celsius array), 1000 times. We generated both discrete and continuous connectivities (K* and K) for all 1000 sample sets, based on the 5% cut-off applied to |r|. We then estimated the eFDR for thresholds (T) of K and K* for all three resources (developing AHBA, adult AHBA and CELSIUS) by calculating the ratio of the mean number of genes that exceeded the threshold T to the observed number of genes in the test set that exceeded T. Examination of the eFDR led to the choice of T that yielded an eFDR = 0.25 as a suitable threshold for determining where to place the cut-off for connectivity.

### Gene prioritization with Endeavour

Finally we compared our prioritization results for the candidate genes to those obtained by an established *in silico* prioritization approach called Endeavour (http://homes.esat.kuleuven.be/~bioiuser/endeavour/index.php) [27]. Endeavour utilizes KEGG, Blast and literature-based resources in addition to non-tissue specific gene expression data for gene prioritization. We compared the rank positions for candidates based on our connectivity scores versus Endeavour's global score and determined Endeavour's score threshold for eFDR = 0.25 using the same approach as that applied to our connectivity scores.

## Results

### Selection of reference and candidate Epileptic Encephalopathy gene sets

The 29 reference Epileptic Encephalopathy genes were all represented on the AHBA gene expression array and in the Celsius resource accessed via UGET.

Of the 182 candidate genes, *C15orf-AP3S2*, *TNNI3K*, *WHSCIL1* were not present on the AHBA array, nor could alternative gene names be found for them, hence they were excluded from the analysis, leaving 179 candidate Epileptic Encephalopathy genes.

Similarly we were unable to identify transcripts representing candidate genes *C15orf-AP3S2*, *LCE1A*, *LDLRAD1*, *MSANTD1*, *OR10S1*, *SGK223*, *SLCO1B7*, *TNNI3K*, *TPTE2*, *WHSC1L1* in the Celsius gene expression data (Affymetrix HG-U133_Plus_2 array), leaving 172 candidate Epileptic Encephalopathy genes to explore in this resource.

Results for Pearson's and Spearman's sample correlation coefficients were very similar but with a loss of power for Spearman's due to the rank transformation. We thus focus on the Pearson's correlation coefficient results, using the Spearman's results as a test for robustness.

### Empirical cumulative distribution function (ECDF) curves for known Epileptic Encephalopathy genes

**Figure 1:**
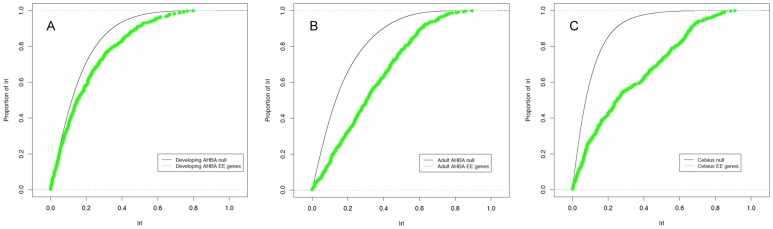
ECDF of Pearson's pairwise correlations shown for 1000 random genes and 29 Epileptic Encephalopathy genes. (A) Developing AHBA (B) Adult AHBA (C) Celsius resource.

The weightings used for each individual to derive both Pearson's and Spearman's correlations for the AHBA data are given in Table S3 and reflect the variability observed for each individual, with lower weights for individuals with greater gene expression correlation variability. The weights are very similar for the two correlation measures. This is not surprising as examination of gene expression of 1000 random genes using a quantile-quantile (Q-Q) plot with theoretical quantiles derived from the normal distribution show a good fit for both the gene expression data and the weighted sums of correlation coefficients for Pearson's correlation coefficient (data not shown). Using Pearson's sample correlation coefficient, we generated ECDF plots for |r| for the 29 reference genes against the null background using expression data from the two AHBA time periods and Celsius (Figure 1). The null distributions (black lines) had median r values of 0.02, 0.05 and −0.01 for the developing AHBA, adult AHBA and Celsius resources respectively (with respective median |r| values of 0.12, 0.13 and 0.07).

The adult AHBA gene expression data showed a greater shift to the right in the ECDF compared to the developing AHBA data suggesting that the reference Epileptic Encephalopathy genes co-express more strongly as a network in adulthood. However, the Celsius resource showed the greatest shift in the ECDF overall. This is reflected in Table 1 with the greatest number of significant connections between Epileptic Encephalopathy genes detected using expression data from Celsius.

**Table 1:** Summary table detailing the |r| 5% significance cut-off values and the number of Epileptic Encephalopathy reference gene pairs reaching this cut-off from a total of n = 406 total gene pairs based on Pearson's correlation coefficient.

Expression data resource	|r| threshold	Number of significant gene pairs (%)
AHBA Developing	0.44	53 (13%)
AHBA Adult	0.48	97 (24%)
Celsius (UGET)	0.30	181 (45%)

### Ordered correlation matrices identifying patterns of co-expression amongst known Epileptic Encephalopathy genes

**Figure 2:**
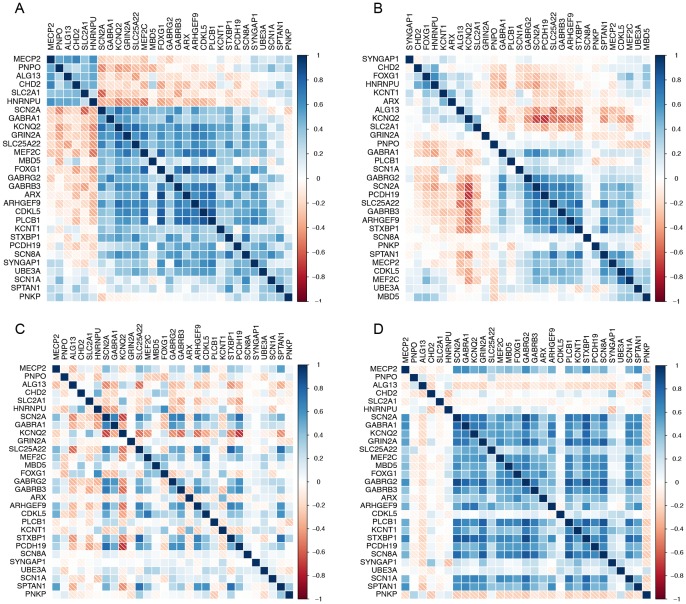
Pairwise Pearson's correlations between pairs of known Epileptic Encephalopathy genes represented as a matrix. (A) Adult AHBA correlation matrix with genes ordered according to the angular distance between the first two principal components. (B) Developing AHBA correlation matrix with genes ordered according to the angular distance between the first two principal components. (C) Developing AHBA correlation matrix with genes ordered according to the angular distance between the first two principal components based on the adult AHBA data. (D) Celsius derived correlation matrix with genes ordered according to the angular distance between the first two principal components of the adult AHBA.

The ordered correlation matrices revealed some striking patterns (Figure 2).

For the adult AHBA data (Figure 2-A) we observed two clusters of positively correlated sets for the 29 known genes. Cluster one is small containing six genes *HNRNPU*, *SLC2A1*, *CHD2*, *ALG13*, *PNPO* and *MECP2*, connected by negative co-expression with the larger cluster containing 23 genes. The average correlation for cluster one is 0.445 and the average correlation for cluster two is 0.380, with an average inter-cluster correlation of −0.100. There are two genes on the fringe of both clusters, *PNKP* and *SPTAN1*, showing weak co-expression with the majority of Epileptic Encephalopathy genes. It is interesting then to note that these genes are both involved in DNA repair. This shared and distinct biological role perhaps can explain their co-expression together and overall relative isolation from the other Epileptic Encephalopathy genes.

The ordered correlation matrix for the developing AHBA data (Figure 2-B) also showed some clustering but it was not as striking as the adult human brain data. This is consistent with the ECDF curves (Figure 1) where the shift in the distribution was not as pronounced. Importantly, when the ordering derived from the clustering of the expression correlations in the adult brain was used, the clustering pattern of two clusters disappeared, and the main cluster correlation was largely eroded (Figure 2-C).

The major adult AHBA cluster of positively correlated genes was recapitulated using the correlation matrix derived from the large, generic gene expression Celsius dataset (Figure 2-D), again consistent with the ECDF shift seen for this dataset (Figure 1). However the second, smaller cluster, containing genes such as *MECP2*, was not detected, suggesting that this is a brain specific signature. Nonetheless both the adult AHBA and Celsius correlation matrices contained clusters of the same co-regulated genes, demonstrating biological replication between two independent gene expression resources. This supports our hypothesis that these 29 known genes show genetic network structure and allows the application of “guilt by association” in prioritizing true Epileptic Encephalopathy genes based on their co-expression with one or more of the genes in this network.

### Comparing candidate Epileptic Encephalopathy gene connectivity scores with other predictive resources

Connectivity scores were generated for each candidate gene. We were interested to compare connectivity scores between those candidates predicted to be pathogenic versus those that were not according to three alternative predictive resources. The permutation test values and standard distribution P-values were very similar for the Mann-Whitney tests, indicating that the lack of independence between data points was not adversely affecting the null distribution. Thus we report the P-values based on the Mann-Whitney distribution.

Discrete (K*) and continuous (K) connectivity scores were significantly higher for candidate genes with gene intolerance scores [28] in the top quartile across all gene expression resources (P-values ranging from 0.012–0.017). In the case of genes that have been previously implicated in other neurological disorders, connectivity scores differed significantly for both the adult AHBA (P-values 0.007 (K*) and 0.006 (K)) and Celsius (P-values 0.006 (K*) and 0.008 (K)) gene expression data results. No significant results were seen between connectivity scores for those genes with variants predicted by PolyPhen-2 [29] to be damaging versus not for any data resource (P-values ranging from 0.060–0.475). Discrete (K*) and continuous (K) connectivity scores were significantly higher for genes with variants whose CADD scores were >25 based on adult AHBA gene expression data only (P-values 0.015 (K*) and 0.013 (K)). In general the discrete connectivity (K*) scores had slightly less power to detect these differences with P-values being slightly larger. Additional Mann-Whitney results are presented in Table S4.

### *In silico* prioritization of candidate Epileptic Encephalopathy genes

A total of 19 genes were prioritized using Pearson's correlation coefficient; seven of these genes were prioritized by more than one of the three gene expression resources utilized. The adult AHBA, developing AHBA and Celsius resource prioritized 4, 10 and 12 candidate genes respectively. These candidates attained the empirical false discovery rate (eFDR) of 0.25 with thresholds of 8.4 (adult AHBA), 7.4 (developing AHBA) and 10.6 (Celsius) using the continuous connectivity measure K only (Figure S1-A, Table 2). Both the developing AHBA and the Celsius resources prioritized *GNAO1*, *RALGPS1*, *ANK3*, *GRIN1* and *MAST1*, with *PLXNA1* prioritized by the developing and adult AHBA resources and *GRIN2B* by the adult AHBA and Celsius.

**Table 2:** The 19 candidate Epileptic Encephalopathy genes prioritized with connectivity measures (K) that met the 0.25 eFDR threshold for at least one of three different gene expression data resources (shown in bold) based on Pearson's correlation coefficient.

Gene	Resource (rank)*	PolyPhen-2	GIT	PE	CADD score >25
*GNAO1*	**Developing AHBA (1)**	Damaging	Intolerant		Yes
	Adult AHBA (32)				
	**Celsius (2)**				
	Endeavour (42)				
*TRIO*	**Developing AHBA (2)**	Damaging	Intolerant		
	Adult AHBA (37)				
	Celsius (49)				
	Endeavour (23)				
*PLXNA1*	**Developing AHBA (3)**		Intolerant		
	**Adult AHBA (4)**				
	Celsius (61)				
	Endeavour (95)				
*RALGPS1*	**Developing AHBA (4)**	Damaging	Intolerant		
	Adult AHBA (74)				
	**Celsius (4)**				
	Endeavour (27)				
*DNM1*	**Developing AHBA (5)**	Damaging	Intolerant		Yes
	Adult AHBA (7)				
	Celsius (16)				
	Endeavour (30)				
*ANK3*	**Developing AHBA (6)**	Damaging	Intolerant		
	Adult AHBA (-)				
	**Celsius (10)**				
	Endeavour (75)				
*IQSEC2*	**Developing AHBA (7)**	Damaging	Intolerant	Yes	Yes
	Adult AHBA (108)				
	Celsius (15)				
	Endeavour (80)				
*GRIN1*	**Developing AHBA (8)**	Damaging	Intolerant	Yes	
	Adult AHBA (23)				
	**Celsius (5)**				
	**Endeavour (3)**				
*MAST1*	**Developing AHBA (9)**		Intolerant		
	Adult AHBA (39)				
	**Celsius (3)**				
	Endeavour (46)				
*PACS2*	**Developing AHBA (10)**	Damaging	Intolerant		
	Adult AHBA (63)				
	Celsius (-)				
	Endeavour (88)				
*KCNB1*	Developing AHBA (16)	Damaging			
	**Adult AHBA (1)**				
	Celsius (14)				
	Endeavour (40)				
*GRIN2B*	Developing AHBA (46)	Damaging	Intolerant	Yes	Yes
	**Adult AHBA (2)**				
	**Celsius (11)**				
	**Endeavour (2)**				
*DAO*	Developing AHBA (-)		Intolerant		
	**Adult AHBA (3)**				
	Celsius (-)				
	Endeavour (65)				
*AKAP6*	Developing AHBA (52)				
	Adult AHBA (48)				
	**Celsius (1)**				
	Endeavour (28)				
*GABRB1*	Developing AHBA (81)	Damaging			Yes
	Adult AHBA (25)				
	**Celsius (6)**				
	**Endeavour (1)**				
*SLC1A2*	Developing AHBA (97)	Damaging	Intolerant		Yes
	Adult AHBA (14)				
	**Celsius (7)**				
	Endeavour (39)				
*YWHAG*	Developing AHBA (13)	Damaging			
	Adult AHBA (15)				
	**Celsius (8)**				
	Endeavour (20)				
*NBEA*	Developing AHBA (22)	Damaging	Intolerant		Yes
	Adult AHBA (13)				
	**Celsius (9)**				
	Endeavour (33)				
*CRTAC1*	Developing AHBA (36)	Damaging	Intolerant		
	Adult AHBA (70)				
	**Celsius (12)**				
	Endeavour (104)				

The single ‘hit’ variants detected in all of these genes were missense changes.

*Rank attained for each data resource (maximum of 179 for the two AHBA resources and a maximum of 172 for Celsius) with a ‘-’ indicating no significant correlations and therefore no possible ranking.

GIT: Gene Intolerance Score.

PE: Prior Evidence for pathological involvement in other neurological disorders.

CADD: Combined Annotation-Dependent Depletion raw score.

The results were very similar for Spearman's correlation coefficient. Again we applied an eFDR of 0.25 to determine a connectivity score threshold for the two AHBA gene expression resources. For the adult AHBA resource, this cutoff resulted in only the top candidate, *KCNB1*, being prioritized (n = 1) and two fewer genes were prioritized with the developing AHBA data (n = 8); six in common with Pearson's (*TRIO*, *GRIN1*, *RALGPS1*, *GNAO1*, *DNM1* and *MAST1*) and two differing (*CEP55* and *SMURF1*). We were unable to apply Spearman's correlation coefficient to the Celsius resource since the data is provided as Pearson's sample correlation coefficients by UGET.

For almost all of these prioritized candidate genes (18 out of 19) there is additional evidence from other sources of information that also implicates them as true Epileptic Encephalopathy genes, such as being predicted damaging by Polyphen-2 or CADD, having a high Gene Intolerance Score or already known to play a role in other neurological disorders such as intellectual disability, autism and malformations of cortical development (Table 2).

Interestingly, the developing AHBA had much greater specificity (more genes prioritized for the same eFDR level chosen) than the adult AHBA, and the Celsius resource in turn shows greater specificity than the developing AHBA with the most number of genes prioritized. This highlights the power of large sample sizes in reducing biological and technical variability and thus detecting signal with a lower false positive rate. See Table S5 for complete set of results for all candidate genes.

### Networks and expression patterns for known and candidate Epileptic Encephalopathy genes

**Figure 3:**
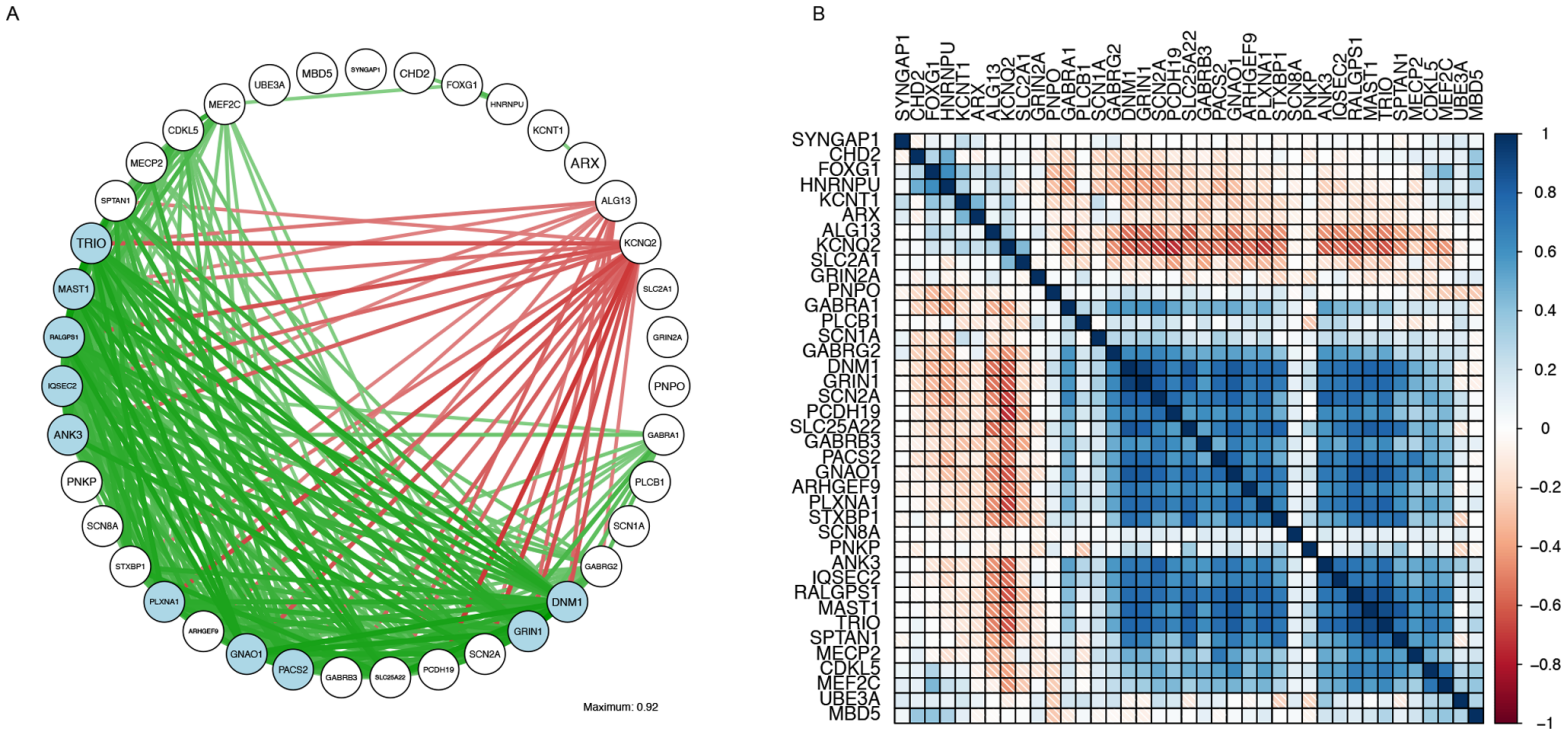
Developing AHBA gene co-expression network and correlation matrix for known and prioritized Epileptic Encephalopathy genes. Gene co-expression networks for the known Epileptic Encephalopathy genes in the top 5% of overall connections of the developing AHBA along with the 10 prioritized candidate Epileptic Encephalopathy genes (shown in blue) as determined by the connectivity measures that are estimated to have an eFDR = 0.25 (thresholded r) using qgraph (A) or represented as an ordered sample correlation (all r values) matrix (B) with ordering based on angular distance. See Figures S2 and S3 for adult AHBA and Celsius results respectively.

Networks of the known and prioritized Epileptic Encephalopathy genes show how highly connected the candidate genes are, commensurate with their prioritization (developing AHBA, Figure 3-A; adult AHBA, Figure S2-A and Celsius, Figure S3-A). Both positive (green) and negative (red) correlations are evident with only those reaching a top 5% cut-off represented graphically.

The clustering observed in the graphical networks also agreed with the ordered sample correlation matrices with similar patterns emerging (developing AHBA, Figure 3-B; adult AHBA, Figure S2-B and Celsius, Figure S3-B).

### Gene prioritization with Endeavour

Endeavour was able to detect all 29 known Epileptic Encephalopathy genes to form a training set in the prioritization of 155 candidate genes (27 candidates were not assessed by Endeavour). Exceeding an eFDR of 0.25 (Figure S1-B), the nine top ranked candidates by Endeavour were *GRIN2B*, *GRIN1*, *GABRB1*, *KCNQ3*, *STK36*, *MYO5A*, *CACNA1A*, *PCDHB13* and *CAMK4*. The Endeavour rank positions for the 19 genes prioritized by our approach are listed in Table 2.

## Discussion

We have shown that 29 known Epileptic Encephalopathy genes show significant co-expression using data from three independent resources, demonstrating important biological replication. In turn we used this information to prioritize a large list of candidate Epileptic Encephalopathy genes based on their co-expression with known causative genes. We demonstrated that our prioritization measure correlated with others that can implicate variants (PolyPhen-2, CADD) or genes (GIT, prior neurological involvement) in disease causality. Confirmation that our prioritization approach works came with two recent publications reporting *GNAO1* [32] and *GRIN2B* [33] as a true Epileptic Encephalopathy genes. Our approach had prioritized *GNAO1* with rank 1 in the developing AHBA and rank 2 in the Celsius resource and *GRIN2B* was ranked 2 with the adult AHBA data and 11 using the Celsius resource (Table 2).

This study has highlighted the strengths and weaknesses of two large-scale gene expression resources, based on markedly different study designs. The Allen Human Brain Atlas (AHBA) is a highly curated, carefully designed study, and although it has many data points (n = 4,904 arrays), these are sampled from only 10 individuals. The resulting data has lower technical variability (reduced artifacts) at the cost of biological variability. Thus this data set represents a lower level of biological and technical variation than that represented by Celsius. However, it is clear from our results that larger sample sizes, such as the thousands of arrays from Celsius, can be beneficial for teasing out true biological signals from technical effects. The advantage of using a brain specific resource, such as the AHBA, is the detection of brain specific signatures that can be incorporated into the *in silico* gene expression analysis providing an even more powerful way of finding relationships between genes. Refinement of the *in silico* prioritization analysis methods should enable better use of smaller sub-networks such as the smaller of the two clusters discovered in the adult AHBA resource. Our analysis is based on a weighted sum of correlation coefficients to derive connectivity, which would benefit from an approach based on principal components, rather than the direct gene-gene correlations [21], representing one such refinement.

Through further analysis of data from the AHBA, we observed for the first time that gene correlation networks for known Epileptic Encephalopathy genes have distinctive brain co-expression patterns at the two very different time periods available (15–20 post-conception weeks versus adulthood). The adult brain shows stronger co-expression signals in comparison to the developing brain, yet prioritizes a smaller number of candidate Epileptic Encephalopathy genes at an equivalent false discovery rate. This suggests much greater variability in the adult brains (derived from just six individuals). The adult brain shows more structured gene co-expression with two gene co-expression modules emerging (Figure 1-A), in comparison to the co-expression networks derived from either of the Celsius or developing AHBA resources. This suggests that more individuals are needed and that currently the large sample sizes of the Celsius resource (N = 5,954) outperform the gain in tissue specificity made by utilizing a brain specific gene expression resource with specificity the highest for this resource (12 Epileptic Encephalopathy genes prioritized at eFDR = 0.25).

Interestingly we have also examined gene expression networks in the AHBA for genes known to be involved in Malformations of Cortical Development and for these we see the developing brain data showing stronger correlation patterns compared to the adult brain (data not shown) representing greater sensitivity. This is consistent with our understanding of when these genes are likely to be of importance (pre-migrational versus post-migrational) and again suggests that disease and time specific resources do add important signal and information.

*In silico* gene prioritization approaches have been promising with many already used in practice. However, the results are variable and real applications with tailored analysis (tissue-specific, with the ability to check data sources) are missing. Popular resource databases such as STRING [34], [35] (http://string-db.org/) show publication-age bias (data not shown), indicative of a strong reliance on text-mining. Addressing this limitation, *in silico* prioritization methods now often include multiple data sources (e.g., Endeavour [27]) but typically remain disease agnostic and are still influenced by text-mining. We were interested to compare our prioritization results with the Endeavour approach and note that only *GRIN2B* of the two now known true positive candidate genes was prioritized (Table 2), whereas our approach prioritized both *GRIN2B* and *GNAO1*. Additionally, our approach was able to explore 179 of the 182 candidate genes (98%) but in comparison only 155 (85%) candidates were available for prioritization by Endeavour, an important limitation for researchers to be aware of when considering *in silico* prioritization methods.

Several *in silico* prioritization methods have been applied to the epilepsy field [36]–[39]. When Chen and colleagues used their method in the familial epilepsy syndrome of Genetic Epilepsy with Febrile Seizures plus (GEFS+), they found that gene expression was the most powerful data source for determining association between five known GEFS+ genes, with little information gained from PPI networks [37]. Consistent with this, Piro and colleagues also considered the known GEFS+ genes and explored an early release of the AHBA, again showing high co-expression between a small reference set of six genes [39]. These studies support our decision to focus on gene expression data and reinforce the notion that unbiased resources are desirable for these types of studies. Some bias still remains in expression array-based resources with only known genes typically represented by array probe sets. This overlooks the many short RNAs which are now gaining greater understanding and promise as candidates for pathogenicity [40]. These array-based resources will be superseded by RNA-seq datasets, promising superior data source options in the future.

Large cohort, massively parallel sequencing studies provide an ideal resource in which to apply *in silico* prioritization, potentially giving an edge to laboratories who can only explore one or a few “best” candidate genes. We have applied *in silico* analysis to putative Epileptic Encephalopathy variant discovery results from recent large-scale studies. Whilst able to show evidence that our prioritization has yielded highly plausible results it is important to note that our findings do not mean that those candidate genes not prioritized should be discounted. It is possible that they represent the first Epileptic Encephalopathy genes in entirely new pathways that would not be discovered using an approach based on known genes. Pragmatically, however, the gathering of functional evidence in support of candidate gene pathogenicity remains costly [11]. This work provides additional support for a small handful of genes that we believe have a stronger case for being true Epileptic Encephalopathy genes and therefore warrant further investment above other candidates.

## Supporting Information

Methods S1 Extended description of methods for weighted correlation matrices and connectivity measures.
(DOCX)

Figure S1 **eFDR estimates as a function (A) of the continuous connectivity (K) for all three gene expression data sets and (B) of Endeavour's 1-Rank scores.** Dotted red line indicates an eFDR = 0.25 with dots near the eFDR plots near 0 to 0.05 indicating the observed connectivities for the top ranked candidate Epileptic Encephalopathy genes for each dataset. The number of discovered variants for each dataset for an eFDR = 0.25 is the number of dots that have been plotted.
(TIFF)

Figure S2 **Adult AHBA gene co-expression network and correlation matrix for known and prioritized Epileptic Encephalopathy genes.** Gene co-expression networks for the known Epileptic Encephalopathy genes that are involved in any of the top 5% of overall connections of the adult AHBA along with the 4 candidate Epileptic Encephalopathy genes (shown in blue) as determined by the connectivity measures that are estimated to have an eFDR = 0.25 (thresholded r) using *qgraph* (A) or represented as an ordered sample correlation (all r values) matrix (B), with ordering based on angular distance.
(TIFF)

Figure S3 **Celsius gene co-expression network and correlation matrix for known and prioritized Epileptic Encephalopathy genes.** Gene co-expression networks for the known Epileptic Encephalopathy genes that are involved in any of the top 5% of overall connections of the Celsius resource along with the 12 candidate Epileptic Encephalopathy genes (shown in blue) as determined by the connectivity measures that are estimated to have an eFDR = 0.25 (thresholded r) using *qgraph* (A) or represented as an ordered sample correlation (all r values) matrix (B), with ordering based on angular distance.
(TIFF)

Table S1 List of known Epileptic Encephalopathy genes chosen from the literature with relevant reference details.
(DOCX)

Table S2 List of 182 candidate Epileptic Encephalopathy genes for prioritization.
(DOCX)

Table S3 Weights derived for each individual AHBA brain for both Pearson's and Spearman's correlation coefficient. The sum of the weights add to one within each of the two time periods.
(DOCX)

Table S4 Extended Mann-Whitney results (P-values). P-values in brackets are those derived using a permutation test with 1000 permutations.
(DOCX)

Table S5 The connectivity (K) and rank position for 179 candidate genes according to the developing AHBA, adult AHBA and Celsius expression data resources and the Endeavour approach. Sheet one shows the results based on Pearson's correlation coefficient and sheet two has Spearman's.
(XLS)
