# Supplementary material for: Harnessing Gene Expression Networks to Prioritize Candidate Epileptic Encephalopathy Genes
Source: PLoS One. 2014 Jul 9;9(7):e102079. doi: 10.1371/journal.pone.0102079 (PMC4090166; doi:10.1371/journal.pone.0102079)
Supplement: Methods S1 — Extended description of methods for weighted correlation matrices and connectivity measures. (DOCX) [file pone.0102079.s001.docx]

**Weighted correlation matrices for the AHBA data**

The pairwise correlations are obtained separately for each of the 6 adult human brains and 4 developing human brains. In order to combine the correlations together, the ‘weighted mean’ method was applied which uses as its weights the inverse sample variance of all of the gene expression values within an individual, summarized by brain structure/array. The weights were determined using the standard normal distribution weighting scheme.

For normally distributed random variates

$$\overline{X}_{W}=\sum_{i=1}^{n} w^{'}_{i}x_{i}=\frac{\sum_{i=1}^{n} w_{i}x_{i}}{\sum_{i=1}^{n} w_{i}},\sum_{i=1}^{n} w^{'}_{i}=1$$

if $X_{i}\sim N(\mu,\sigma_{i}^{2})$ then

$$w_{i}=\frac{1}{\sigma^{2}_{i}}$$

Using the Pearson’s sample correlation coefficients, derived for each gene pair within each individual, we estimate the weights based on the distribution of the sample correlation coefficients within each individual.

Thus,

where n is the number of gene pairs on the gene expression array and

$r_{k,l}^{i}$is the Pearson’s sample correlation coefficient in the ith individual for gene pair (k,l), and $\bar{r}^{i}$is the mean sample correlation coefficient for individual i.

Visual inspection of the sample distribution of r within each individual verified approximate normality.

**Definitions of Connectivity**

We define two measures of connectivity. Let K_i_= connectivity of candidate gene i, measured as the absolute value of the correlation coefficients to each member gene of our network of known genes Q. K_i_^*^ is the discretized version.

where

$$K^{*}_{i}=\sum_{j\in Q} I(|r_{i,j}|\text{ }>T)$$
