# Supplementary figures and images for: Harnessing Gene Expression Networks to Prioritize Candidate Epileptic Encephalopathy Genes
Source: PLoS One. 2014 Jul 9;9(7):e102079. doi: 10.1371/journal.pone.0102079 (PMC4090166; doi:10.1371/journal.pone.0102079)

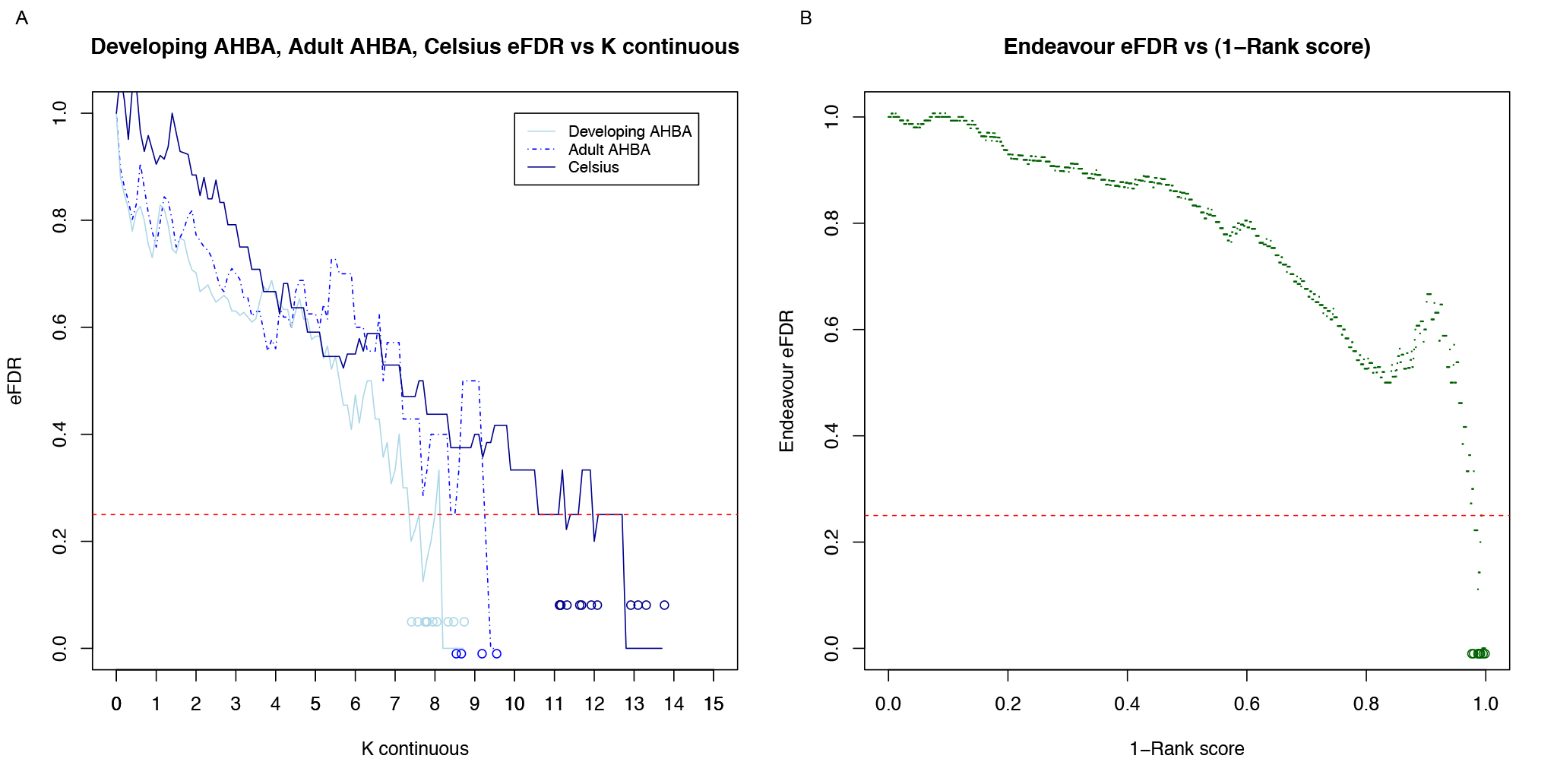

Supplement: Figure S1 — eFDR estimates as a function (A) of the continuous connectivity (K) for all three gene expression data sets and (B) of Endeavour's 1-Rank scores. Dotted red line indicates an eFDR = 0.25 with dots near the eFDR plots near 0 to 0.05 indicating the observed connectivities for the top ranked candidate Epileptic Encephalopathy genes for each dataset. The number of discovered variants for each dataset for an eFDR = 0.25 is the number of dots that have been plotted. (TIFF) [file pone.0102079.s002.tif]

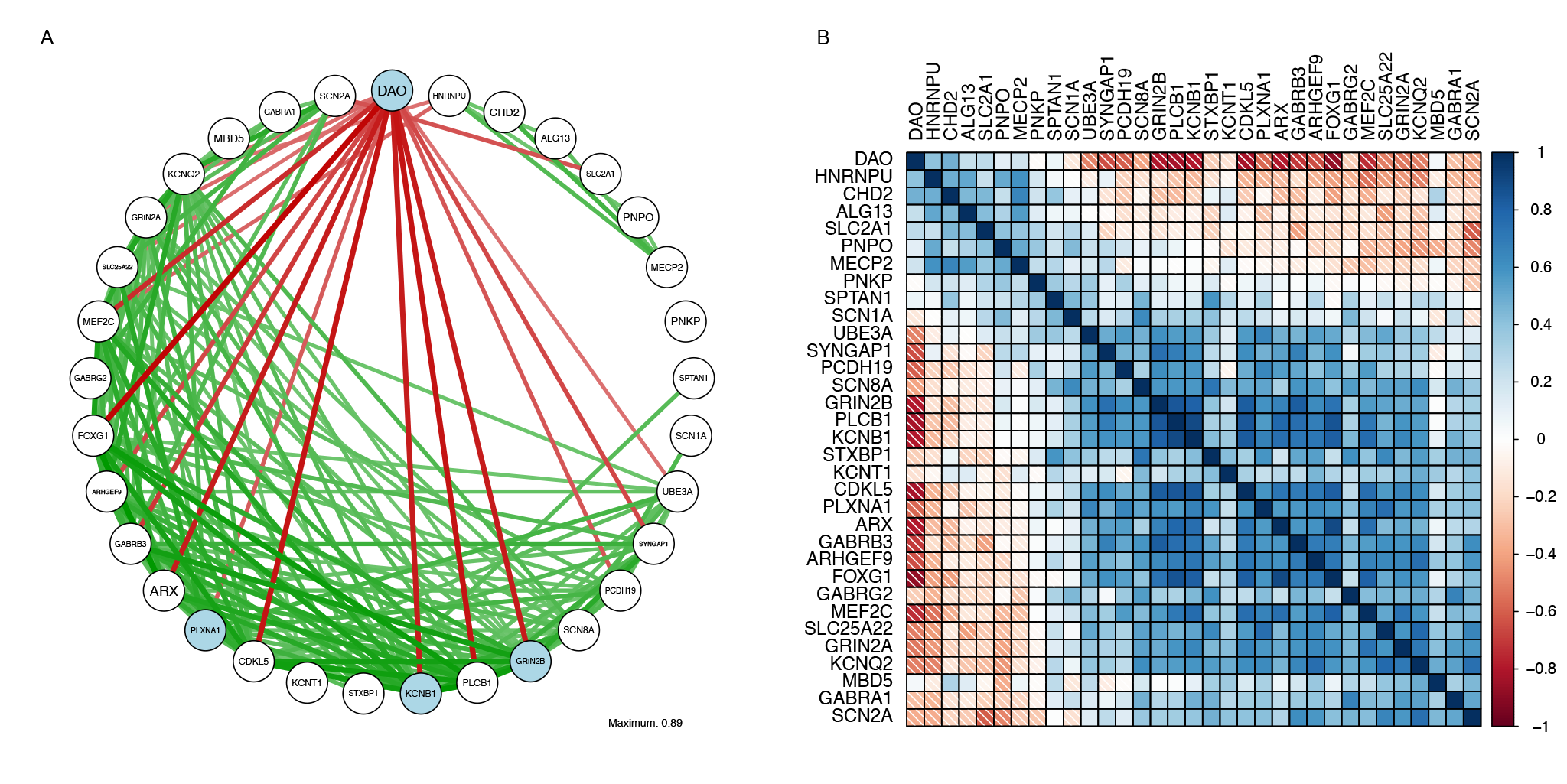

Supplement: Figure S2 — Adult AHBA gene co-expression network and correlation matrix for known and prioritized Epileptic Encephalopathy genes. Gene co-expression networks for the known Epileptic Encephalopathy genes that are involved in any of the top 5% of overall connections of the adult AHBA along with the 4 candidate Epileptic Encephalopathy genes (shown in blue) as determined by the connectivity measures that are estimated to have an eFDR = 0.25 (thresholded r) using qgraph (A) or represented as an ordered sample correlation (all r values) matrix (B), with ordering based on angular distance. (TIFF) [file pone.0102079.s003.tif]

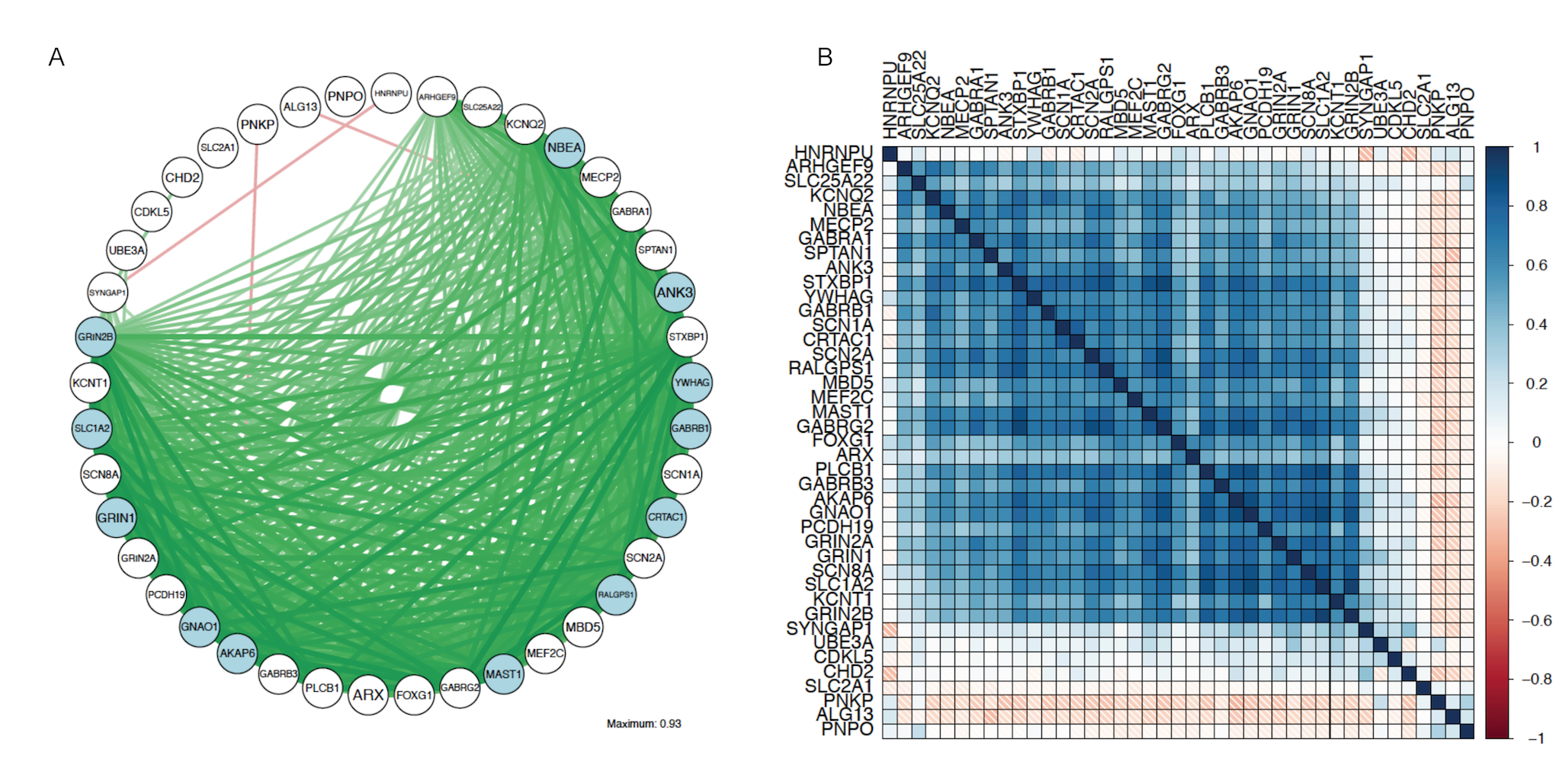

Supplement: Figure S3 — Celsius gene co-expression network and correlation matrix for known and prioritized Epileptic Encephalopathy genes. Gene co-expression networks for the known Epileptic Encephalopathy genes that are involved in any of the top 5% of overall connections of the Celsius resource along with the 12 candidate Epileptic Encephalopathy genes (shown in blue) as determined by the connectivity measures that are estimated to have an eFDR = 0.25 (thresholded r) using qgraph (A) or represented as an ordered sample correlation (all r values) matrix (B), with ordering based on angular distance. (TIFF) [file pone.0102079.s004.tif]
