# Supplementary material for: Harnessing Gene Expression Networks to Prioritize Candidate Epileptic Encephalopathy Genes
Source: PLoS One. 2014 Jul 9;9(7):e102079. doi: 10.1371/journal.pone.0102079 (PMC4090166; doi:10.1371/journal.pone.0102079)
Supplement: Table S1 — List of known Epileptic Encephalopathy genes chosen from the literature with relevant reference details. (DOCX) [file pone.0102079.s005.docx]

| **Gene Name** | **Reference(s)** |
| --- | --- |
| *ALG13* | [[1](#_ENREF_1)] |
| *ARHGEF9* | [[2](#_ENREF_2)] |
| *ARX* | [[3](#_ENREF_3)] |
| *CDKL5* | [[4](#_ENREF_4)] |
| *CHD2* | [[5](#_ENREF_5)] |
| *FOXG1* | [[6](#_ENREF_6)] |
| *GABRA1* | [1,7] |
| *GABRB3* | [[1](#_ENREF_1)] |
| *GABRG2* | [5,8] |
| *GRIN2A* | [9,10,11] |
| *HNRNPU* | [[1](#_ENREF_1),[5](#_ENREF_5)] |
| *KCNQ2* | [12] |
| *KCNT1* | [13] |
| *MBD5* | [5,14] |
| *MECP2* | [15] |
| *MEF2C* | [5,16,17] |
| *PCDH19* | [18] |
| *PLCB1* | [19,20] |
| *PNKP* | [21] |
| *PNPO* | [22] |
| *SCN1A* | [23] |
| *SCN2A* | [24,25] |
| *SCN8A* | [1,5,26] |
| *SLC2A1* | [27] |
| *SLC25A22* | [28,29] |
| *SPTAN1* | [30] |
| *STXBP1* | [31] |
| *SYNGAP1* | [[5](#_ENREF_5)] |
| *UBE3A* | [32,33] |

**Supplemental References**

1. EPi4K Consortium, Epilepsy Phenome/Genome Project, Allen AS, Berkovic SF, Cossette P, et al. (2013) De novo mutations in epileptic encephalopathies. Nature.

2. Harvey K, Duguid IC, Alldred MJ, Beatty SE, Ward H, et al. (2004) The GDP-GTP exchange factor collybistin: an essential determinant of neuronal gephyrin clustering. J Neurosci 24: 5816-5826.

3. Stromme P, Mangelsdorf ME, Shaw MA, Lower KM, Lewis SM, et al. (2002) Mutations in the human ortholog of Aristaless cause X-linked mental retardation and epilepsy. Nat Genet 30: 441-445.

4. Kalscheuer VM, Tao J, Donnelly A, Hollway G, Schwinger E, et al. (2003) Disruption of the serine/threonine kinase 9 gene causes severe X-linked infantile spasms and mental retardation. Am J Hum Genet 72: 1401-1411.

5. Carvill GL, Heavin SB, Yendle SC, McMahon JM, O'Roak BJ, et al. (2013) Targeted resequencing in epileptic encephalopathies identifies de novo mutations in CHD2 and SYNGAP1. Nat Genet 45: 825-830.

6. Ariani F, Hayek G, Rondinella D, Artuso R, Mencarelli MA, et al. (2008) FOXG1 is responsible for the congenital variant of Rett syndrome. Am J Hum Genet 83: 89-93.

7. Carvill GL, Weckhuysen S, McMahon JM, Hartmann C, Moller RS, et al. (in press) GABRA1 and STXBP1: novel genetic causes of Dravet syndrome. Neurology.

8. Singh R, Scheffer IE, Crossland K, Berkovic SF (1999) Generalized epilepsy with febrile seizures plus: a common childhood-onset genetic epilepsy syndrome. Ann Neurol 45: 75-81.

9. Carvill GL, Regan BM, Yendle SC, O'roak BJ, Lozovaya N, et al. (2013) GRIN2A mutations cause epilepsy-aphasia spectrum disorders. Nature Genetics.

10. Lemke JR, Lal D, Reinthaler EM, Steiner I, Nothnagel M, et al. (2013) Mutations in GRIN2A cause idiopathic focal epilepsy with rolandic spikes. Nature Genetics.

11. Lesca G, Rudolf G, Bruneau N, Lozovaya N, Labalme A, et al. (2013) GRIN2A mutations in acquired epileptic aphasia and related childhood focal epilepsies and encephalopathies with speech and language dysfunction. Nature Genetics.

12. Weckhuysen S, Mandelstam S, Suls A, Audenaert D, Deconinck T, et al. (2012) KCNQ2 encephalopathy: emerging phenotype of a neonatal epileptic encephalopathy. Ann Neurol 71: 15-25.

13. Barcia G, Fleming MR, Deligniere A, Gazula VR, Brown MR, et al. (2012) De novo gain-of-function KCNT1 channel mutations cause malignant migrating partial seizures of infancy. Nat Genet 44: 1255-1259.

14. Talkowski ME, Mullegama SV, Rosenfeld JA, van Bon BW, Shen Y, et al. (2011) Assessment of 2q23.1 microdeletion syndrome implicates MBD5 as a single causal locus of intellectual disability, epilepsy, and autism spectrum disorder. Am J Hum Genet 89: 551-563.

15. Amir RE, Van den Veyver IB, Wan M, Tran CQ, Francke U, et al. (1999) Rett syndrome is caused by mutations in X-linked MECP2, encoding methyl-CpG-binding protein 2. Nat Genet 23: 185-188.

16. Le Meur N, Holder-Espinasse M, Jaillard S, Goldenberg A, Joriot S, et al. (2010) MEF2C haploinsufficiency caused by either microdeletion of the 5q14.3 region or mutation is responsible for severe mental retardation with stereotypic movements, epilepsy and/or cerebral malformations. J Med Genet 47: 22-29.

17. Zweier M, Gregor A, Zweier C, Engels H, Sticht H, et al. (2010) Mutations in MEF2C from the 5q14.3q15 microdeletion syndrome region are a frequent cause of severe mental retardation and diminish MECP2 and CDKL5 expression. Hum Mutat 31: 722-733.

18. Dibbens LM, Tarpey PS, Hynes K, Bayly MA, Scheffer IE, et al. (2008) X-linked protocadherin 19 mutations cause female-limited epilepsy and cognitive impairment. Nat Genet 40: 776-781.

19. Kurian MA, Meyer E, Vassallo G, Morgan NV, Prakash N, et al. (2010) Phospholipase C beta 1 deficiency is associated with early-onset epileptic encephalopathy. Brain 133: 2964-2970.

20. Poduri A, Chopra SS, Neilan EG, Elhosary PC, Kurian MA, et al. (2012) Homozygous PLCB1 deletion associated with malignant migrating partial seizures in infancy. Epilepsia 53: e146-150.

21. Shen J, Gilmore EC, Marshall CA, Haddadin M, Reynolds JJ, et al. (2010) Mutations in PNKP cause microcephaly, seizures and defects in DNA repair. Nat Genet 42: 245-249.

22. Mills PB, Surtees RA, Champion MP, Beesley CE, Dalton N, et al. (2005) Neonatal epileptic encephalopathy caused by mutations in the PNPO gene encoding pyridox(am)ine 5'-phosphate oxidase. Hum Mol Genet 14: 1077-1086.

23. Claes L, Del-Favero J, Ceulemans B, Lagae L, Van Broeckhoven C, et al. (2001) De novo mutations in the sodium-channel gene SCN1A cause severe myoclonic epilepsy of infancy. Am J Hum Genet 68: 1327-1332.

24. Kamiya K, Kaneda M, Sugawara T, Mazaki E, Okamura N, et al. (2004) A nonsense mutation of the sodium channel gene SCN2A in a patient with intractable epilepsy and mental decline. J Neurosci 24: 2690-2698.

25. Ogiwara I, Ito K, Sawaishi Y, Osaka H, Mazaki E, et al. (2009) De novo mutations of voltage-gated sodium channel alphaII gene SCN2A in intractable epilepsies. Neurology 73: 1046-1053.

26. Veeramah KR, O'Brien JE, Meisler MH, Cheng X, Dib-Hajj SD, et al. (2012) De novo pathogenic SCN8A mutation identified by whole-genome sequencing of a family quartet affected by infantile epileptic encephalopathy and SUDEP. Am J Hum Genet 90: 502-510.

27. Seidner G, Alvarez MG, Yeh JI, O'Driscoll KR, Klepper J, et al. (1998) GLUT-1 deficiency syndrome caused by haploinsufficiency of the blood-brain barrier hexose carrier. Nat Genet 18: 188-191.

28. Molinari F, Kaminska A, Fiermonte G, Boddaert N, Raas-Rothschild A, et al. (2009) Mutations in the mitochondrial glutamate carrier SLC25A22 in neonatal epileptic encephalopathy with suppression bursts. Clin Genet 76: 188-194.

29. Molinari F, Raas-Rothschild A, Rio M, Fiermonte G, Encha-Razavi F, et al. (2005) Impaired mitochondrial glutamate transport in autosomal recessive neonatal myoclonic epilepsy. Am J Hum Genet 76: 334-339.

30. Saitsu H, Tohyama J, Kumada T, Egawa K, Hamada K, et al. (2010) Dominant-negative mutations in alpha-II spectrin cause West syndrome with severe cerebral hypomyelination, spastic quadriplegia, and developmental delay. Am J Hum Genet 86: 881-891.

31. Saitsu H, Kato M, Mizuguchi T, Hamada K, Osaka H, et al. (2008) De novo mutations in the gene encoding STXBP1 (MUNC18-1) cause early infantile epileptic encephalopathy. Nat Genet 40: 782-788.

32. Kishino T, Lalande M, Wagstaff J (1997) UBE3A/E6-AP mutations cause Angelman syndrome. Nat Genet 15: 70-73.

33. Matsuura T, Sutcliffe JS, Fang P, Galjaard RJ, Jiang YH, et al. (1997) De novo truncating mutations in E6-AP ubiquitin-protein ligase gene (UBE3A) in Angelman syndrome. Nat Genet 15: 74-77.
