# Supplementary material for: Harnessing Gene Expression Networks to Prioritize Candidate Epileptic Encephalopathy Genes
Source: PLoS One. 2014 Jul 9;9(7):e102079. doi: 10.1371/journal.pone.0102079 (PMC4090166; doi:10.1371/journal.pone.0102079)
Supplement: Table S2 — List of 182 candidate Epileptic Encephalopathy genes for prioritization. (DOCX) [file pone.0102079.s006.docx]

| *AKR1C4* | *MTOR* | *ACOT4* | *SAFB2* |
| --- | --- | --- | --- |
| *ALMS1* | *MYH6* | *AGPAT3* | *SKA3* |
| *ANK3* | *MYO3A* | *AHCY* | *SLCO1B7* |
| *ANKRD12* | *NCOR2* | *AKAP6* | *SNX30* |
| *ASXL1* | *NEDD4L* | *CDS2* | *STK36* |
| *ATP2B4* | *NFASC* | *CELA3B* | *THAP4* |
| *BEST2* | *NLGN2* | *CEP55* | *TPTE2* |
| *C17orf53* | *NLRP5* | *CHIA* | *UNC5CL* |
| *C18orf25* | *NLRP8* | *COQ3* | *XPO1* |
| *C1QTNF6* | *NOTUM* | *CYP2U1* | *ZNF839* |
| *CACNA1A* | *NR1H2* | *DAO* | *COL4A4* |
| *CHD4* | *OR10S1* | *DIP2C* | *COL7A1* |
| *CNTN5* | *PACS2* | *DTYMK* | *LCE1A* |
| *CR2* | *PALLD* | *EXOSC2* | *SCAF4* |
| *CRTAC1* | *PCDHB13* | *FCGR2B* | *TTN* |
| *CSMD2* | *PDIK1L* | *FLNC* | *ZFHX3* |
| *CSNK1E* | *PIK3AP1* | *FRAT2* | *IQSEC2* |
| *CUX2* | *PLA1A* | *GCM2* | *MMP27* |
| *DDX50* | *PNMAL1* | *GPR108* | *IFT172* |
| *DDX58* | *PRDM12* | *GPR98* | *CELSR1* |
| *DHDDS* | *PTPRR* | *GRAMD2* | *DCX* |
| *DHTKD1* | *RARS* | *HBS1L* | *FAM63B* |
| *DIAPH3* | *RFX3* | *HCK* | *MYO5A* |
| *DNAH7* | *RIOK3* | *HIST1H2BD* | *NBEA* |
| *DNM1* | *RRP1B* | *HRG* | *RAB5C* |
| *DPP7* | *RTKN2* | *HSF2* | *RALGAPB* |
| *FAM102A* | *RXFP1* | *IQSEC1* | *RGS14* |
| *FAM116B* | *SDCBP2* | *KLHL11* | *RYR2* |
| *FAM50A* | *SGK223* | *KRT34* | *SLC35A2* |
| *FAM86C1* | *SLAMF1* | *KRTAP1-3* | *VPS37A* |
| *FASN* | *SLC16A3* | *LDLRAD1* | *C15orf38-AP3S2* |
| *FLNA* | *SLC1A2* | *MAPK8IP1* | *ALS2CL* |
| *GABRB1* | *SMG9* | *MAST1* | *C16orf62* |
| *GAS2* | *SMURF1* | *MKLN1* | *C3orf22* |
| *GLB1L3* | *SPG7* | *MLL4* | *C4orf37* |
| *GNAO1* | *TAK1* | *MTRF1* | *CAMK4* |
| *GPR128* | *THOC2* | *MYO7B* | *CCDC125* |
| *GRIN2B* | *TIFA* | *NFE2L1* | *CDC25B* |
| *GRIN1* | *TNN13K* | *OSBPL5* |  |
| *HIPK3* | *TRIM29* | *PLXNA1* |  |
| *ITGB4* | *TRIO* | *PRKX* |  |
| *KCNB1* | *TRRAP* | *PRR19* |  |
| *KCNQ3* | *TSNAXIP1* | *PTEN* |  |
| *KIAA1324L* | *WDR19* | *PWWP2A* |  |
| *KIAA2018* | *WHSC1L1* | *RALGPS1* |  |
| *MEOX2* | *YWHAG* | *RANGAP1* |  |
| *MLL* | *ZSCAN2* | *RTP1* |  |
| *MSANTD1* | *ABCB9* | *RUVBL2* |  |
