# Supplementary material for: Harnessing Gene Expression Networks to Prioritize Candidate Epileptic Encephalopathy Genes
Source: PLoS One. 2014 Jul 9;9(7):e102079. doi: 10.1371/journal.pone.0102079 (PMC4090166; doi:10.1371/journal.pone.0102079)
Supplement: Table S3 — Weights derived for each individual AHBA brain for both Pearson's and Spearman's correlation coefficient. The sum of the weights add to one within each of the two time periods. (DOCX) [file pone.0102079.s007.docx]

| **Individual** | **Time Period** | **Sex** | **Number of arrays** | **Pearson’s weight** | **Spearman’s weight** |
| --- | --- | --- | --- | --- | --- |
| 9861 | Adult | Male | 946 | 0.23 | 0.22 |
| 10021 | Adult | Male | 893 | 0.16 | 0.15 |
| 12876 | Adult | Male | 363 | 0.18 | 0.18 |
| 14380 | Adult | Male | 529 | 0.14 | 0.14 |
| 15496 | Adult | Female | 470 | 0.15 | 0.16 |
| 15697 | Adult | Male | 501 | 0.15 | 0.16 |
| 12566 | Developing | Male | 310 | 0.26 | 0.26 |
| 12690 | Developing | Female | 226 | 0.25 | 0.25 |
| 12840 | Developing | Female | 326 | 0.23 | 0.23 |
| 14751 | Developing | Female | 340 | 0.26 | 0.26 |
