# Supplementary material for: Harnessing Gene Expression Networks to Prioritize Candidate Epileptic Encephalopathy Genes
Source: PLoS One. 2014 Jul 9;9(7):e102079. doi: 10.1371/journal.pone.0102079 (PMC4090166; doi:10.1371/journal.pone.0102079)
Supplement: Table S4 — Extended Mann-Whitney results (P-values). P-values in brackets are those derived using a permutation test with 1000 permutations. (DOCX) [file pone.0102079.s008.docx]

Pearson

| **Dataset** | **Intolerance** | | **Prior neuro gene** | | **PolyPhen-2** | | **CADD score** | |
| --- | --- | --- | --- | --- | --- | --- | --- | --- |
|  | **K*** | **K** | **K*** | **K** | **K*** | **K** | **K*** | **K** |
| AHB | 0.017  (0.016) | 0.015  (0.012) | 0.007  (0.004) | 0.006  (0.004) | 0.060  (0.055) | 0.067  (0.063) | 0.015  (0.017) | 0.013  (0.014) |
| DHB | 0.012  (0.013) | 0.017  (0.016) | 0.249  (0.265) | 0.289  (0.302) | 0.281  (0.282) | 0.339  (0.342) | 0.187  (0.211) | 0.192  (0.221) |
| CELSIUS | 0.016  (0.017) | 0.016  (0.017) | 0.006  (0.006) | 0.008  (0.011) | 0.469  (0.453) | 0.475  (0.462) | 0.405  (0.386) | 0.454  (0.433) |

Spearman

| **Dataset** | **Intolerance** | | **Prior neuro gene** | | **PolyPhen-2** | | **CADD score** | |
| --- | --- | --- | --- | --- | --- | --- | --- | --- |
|  | **K*** | **K** | **K*** | **K** | **K*** | **K** | **K*** | **K** |
| AHB | 0.016  (0.017) | 0.011  (0.011) | 0.009  (0.006) | 0.010  (0.008) | 0.050  (0.054) | 0.071  (0.074) | 0.019  (0.017) | 0.028  0.030 |
| DHB | 0.018  (0.019) | 0.023  (0.022) | 0.227  (0.216) | 0.263  (0.250) | 0.339  (0.320) | 0.390  (0.377) | 0.189  (0.214) | 0.205  (0.228) |

Endeavour

| **Dataset** | **Intolerance** | **Prior neuro gene** | **PolyPhen-2** | **CADD score** |
| --- | --- | --- | --- | --- |
| Endeavour | 0.038  (0.034) | 7.2x10^-5^  (0.001) | 0.120  (0.122) | 8.4x10^-4^  (0.001) |
